# Supplementary figures and images for: Axons compensate for biophysical constraints of variable size to uniformize their action potentials
Source: PLoS Biol. 2024 Dec 2;22(12):e3002929. doi: 10.1371/journal.pbio.3002929 (PMC11637306; doi:10.1371/journal.pbio.3002929)

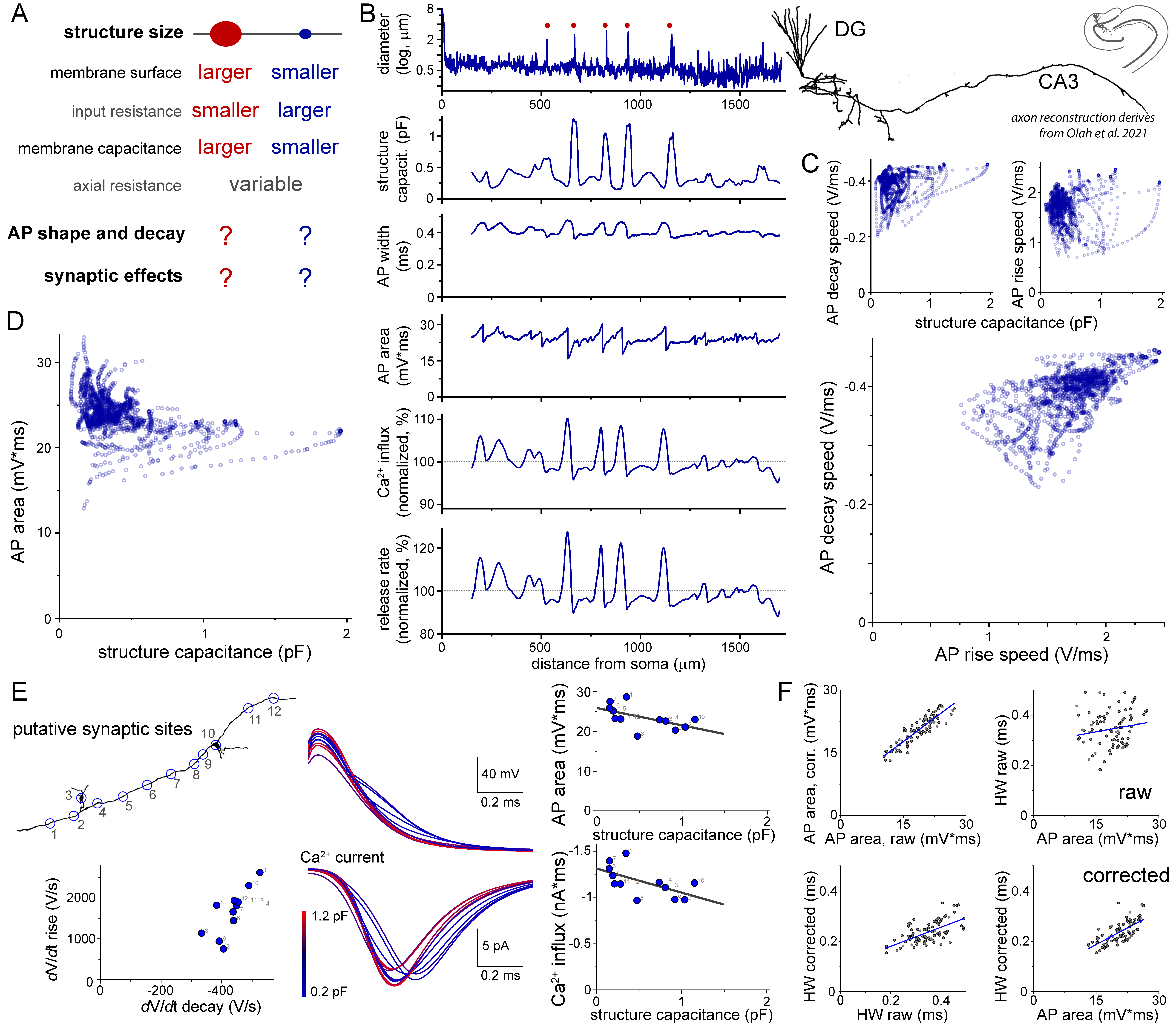

Supplement: S1 Fig — (A) Theoretical rules—derived from passive RC properties of neuronal membranes—defining the dependence of the speed of neuronal signals, presumably including axonal AP shape, on the size of the structure. (B) APs were simulated in a 3D reconstructed mossy fiber by using homogeneously distributed Hodgkin–Huxley sodium and potassium conductances and shown along the axon with variable diameter. Only the data from the middle section of the axon is shown. Ca2+ influx was simulated by using a realistic set of Ca2+ conductance [73]. Synaptic release related to the integrated Ca2+ influx on the 2.5× power. (C) Variable AP kinetics measured as rate of rise and decay depending on the biophysical size of simulated axon compartment. The rise and decay phases of APs show the expected correlation at population level; however, this correlation is variable between individual boutons due to the influence of neighboring axonal regions. (D) Area of simulated APs along the entire axon demonstrate the large variability and axon size-dependence of AP shape. Simulated APs were measured at every 1 μm and were plotted against the membrane capacitance of the same spots. AP area was the integral of the repolarization phase only to avoid contamination of the measurement by effects on the rise phase of the APs [17–20]. (E) AP area and simulated Ca2+ influx at 12 boutons that potentially form synaptic contacts within a short segment (linear fits AP area: R2: 0.249, ANOVA p > F: 0.057, slope: −4.37. ICa: R2: 0.287 ANOVA p > F 0.042, slope: 0.261). Colors of traces scales with the capacitance of the boutons. (F) To demonstrate the reliability of AP area measurements compared to classical AP kinetic parameters, we compared raw electrophysiological recordings of APs with their corrected waveforms (see Fig 1F). The correlation between the half-width of APs (HW) and AP area was weaker in raw AP measurements compared to the corrected APs (R2: 0.389 vs. 0.814, left panels). Furthermore, in theory, HW an [file pbio.3002929.s001.tif]

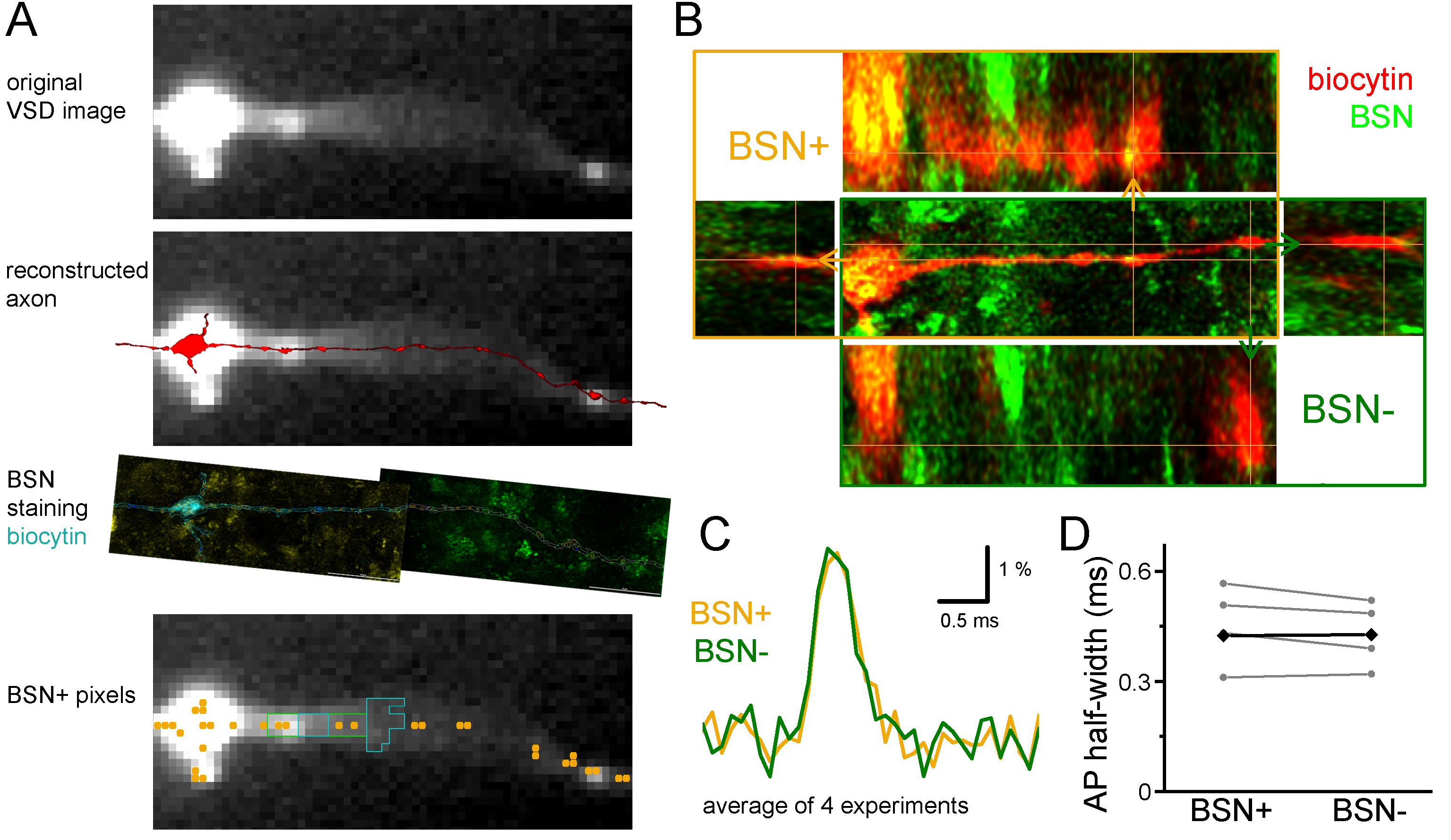

Supplement: S2 Fig — (A) Synaptic regions were identified by presynaptic marker, bassoon (BSN) positivity. Individual pixels were assigned by matching the post hoc reconstruction of biocytin labeled segments that were subject of VSD imaging. Only neighboring segments were included to avoid the contamination by conduction delay. LMFBs were excluded. (B) Confocal image analysis of the bassoon puncta in the biocytin labeled axon. (C) Average APs from 4 experiments with correlated synapse identification. (D) Comparison of the half-widths of APs from sMF segments identified as synaptic or non-synaptic based on BSN staining. Source data are available at https://repo.researchdata.hu/dataset.xhtml?persistentId=hdl:21.15109/ARP/BTK8A4. (TIF) [file pbio.3002929.s002.tif]

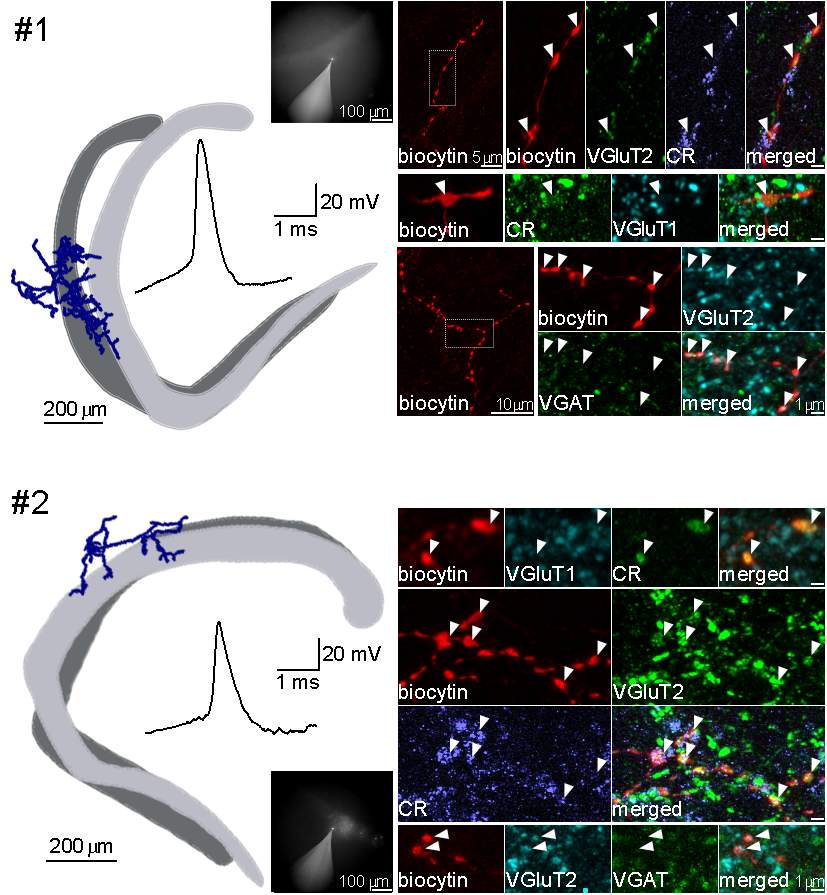

Supplement: S3 Fig — Two examples are shown with the reconstructions of the biocytin-labeled axons and immunolabeling for typical SuM markers and proteins that are present in other axons. SuM axons restricted to the granule cell layer and inner moleculare layer of the DG (3D-represented by light and dark gray areas). Original images are available at https://repo.researchdata.hu/dataset.xhtml?persistentId=hdl:21.15109/ARP/BTK8A4. (TIF) [file pbio.3002929.s003.tif]

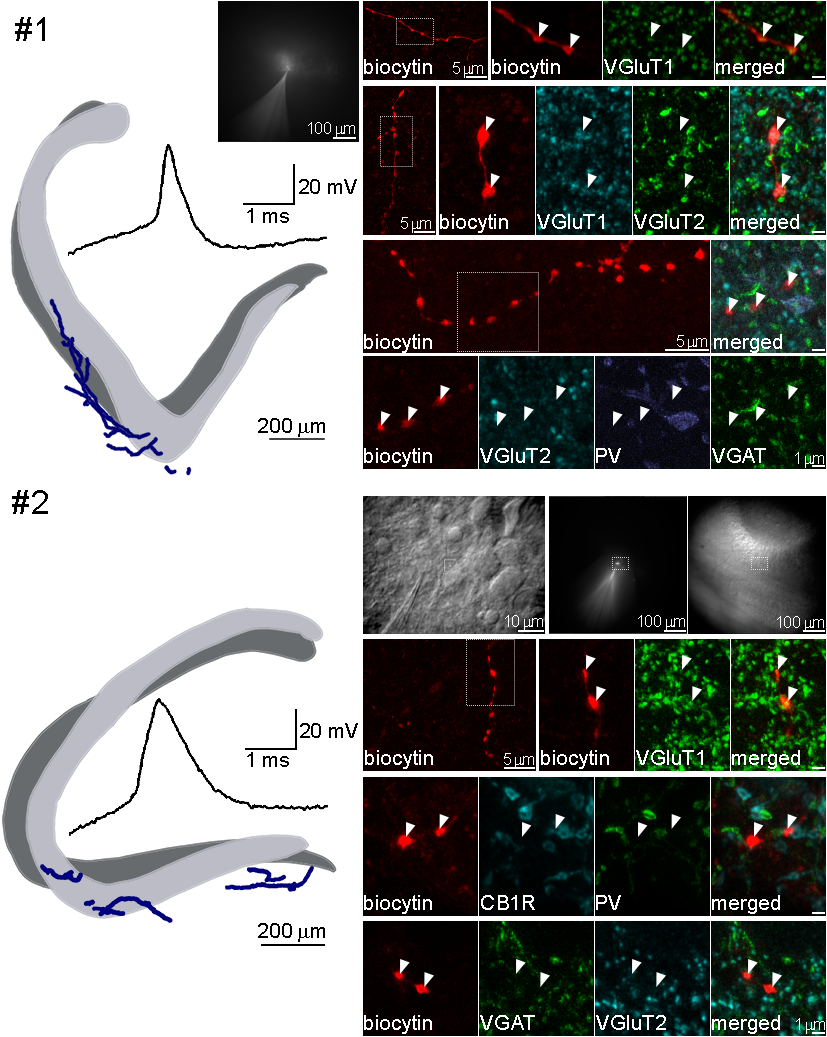

Supplement: S4 Fig — Two examples are shown with the reconstructions of the biocytin-labeled axons and immunolabeling for typical MC markers and proteins that are present in other axons. MC axons restricted to the inner molecular layer of the DG. Original images are available at https://repo.researchdata.hu/dataset.xhtml?persistentId=hdl:21.15109/ARP/BTK8A4. (TIF) [file pbio.3002929.s004.tif]

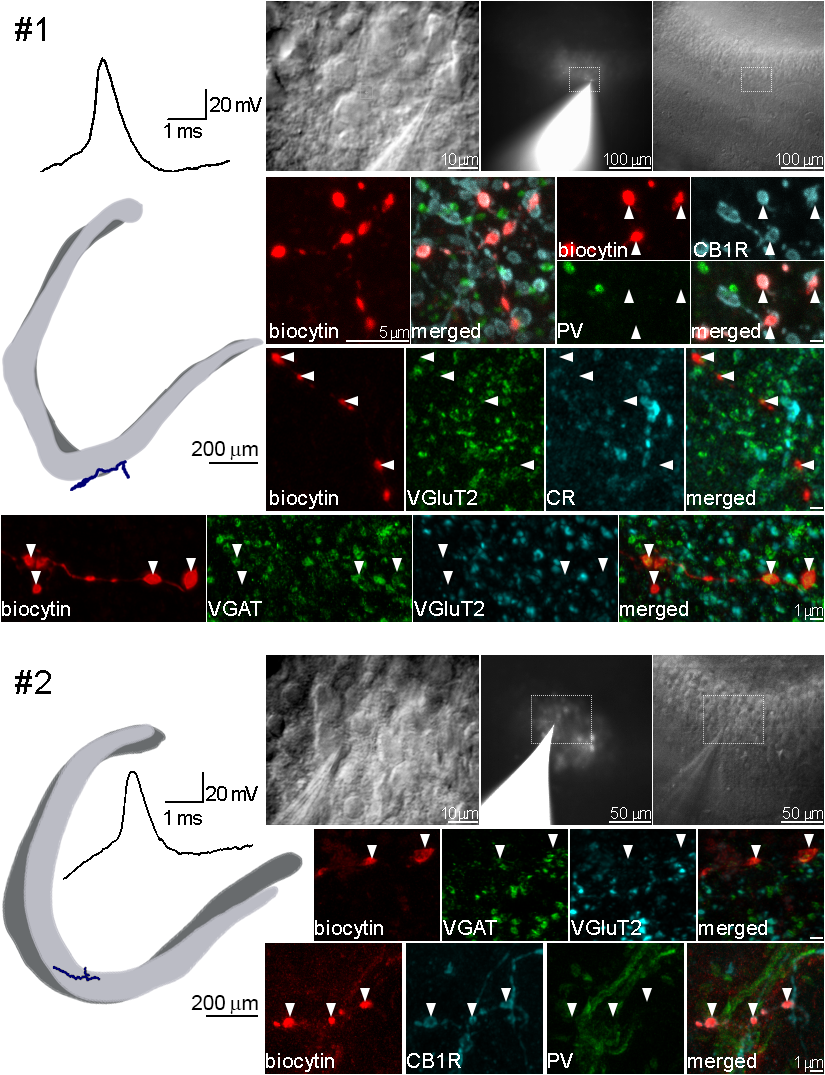

Supplement: S5 Fig — Two examples are shown with the reconstructions of the biocytin-labeled axons and immunolabeling for typical GABAergic cell markers and proteins that are present in other axons. Original images are available at https://repo.researchdata.hu/dataset.xhtml?persistentId=hdl:21.15109/ARP/BTK8A4. (TIF) [file pbio.3002929.s005.tif]

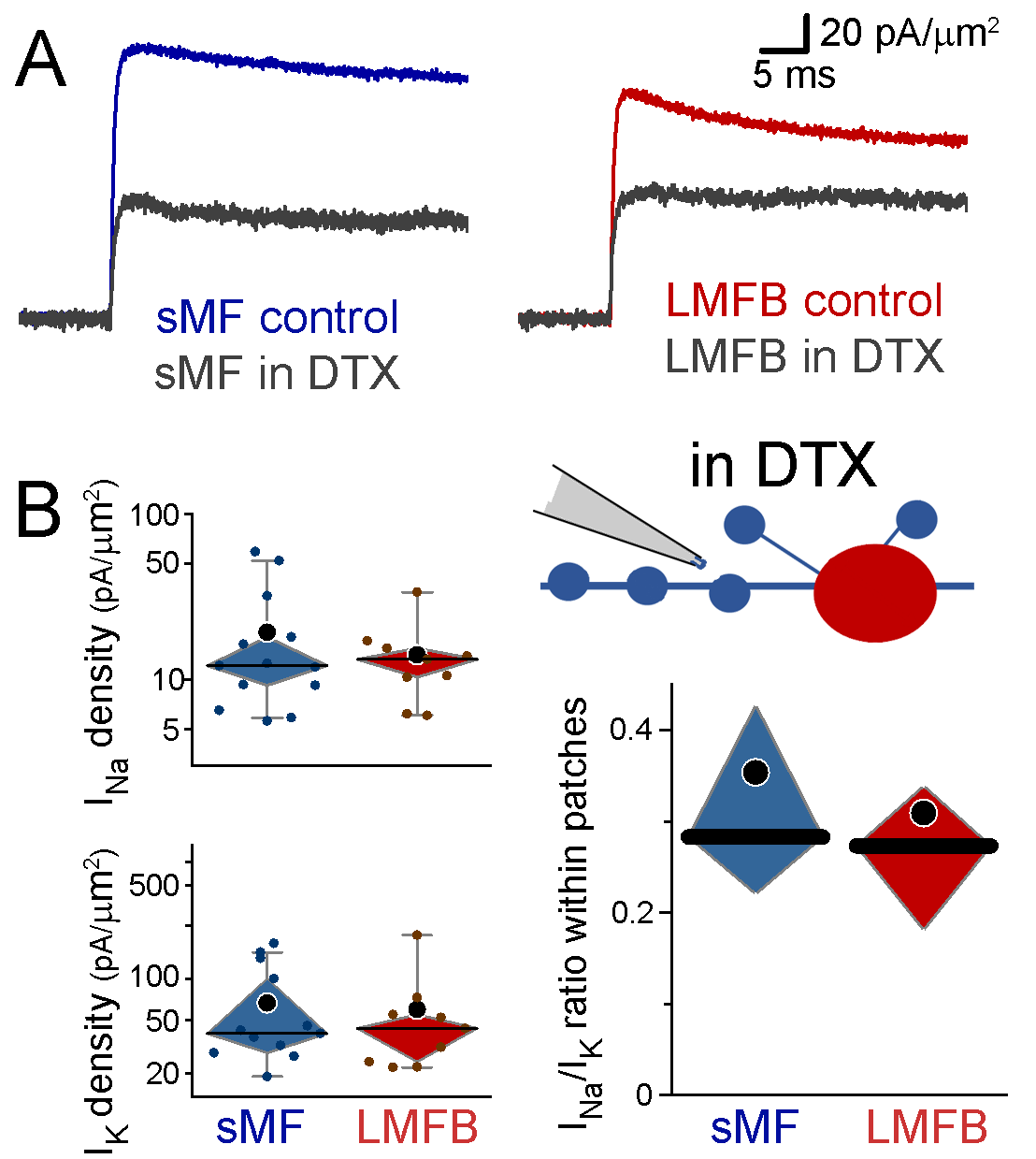

Supplement: S6 Fig — (A) Average of IK current densities in membrane patches pulled from sMFs and LMFBs in the presence of DTX (gray traces) and in control conditions. (B) The average INa and IK densities and INa/IK ratio were similar in LMFB and sMF recordings in the presence of DTX. Source data are available at https://repo.researchdata.hu/dataset.xhtml?persistentId=hdl:21.15109/ARP/BTK8A4. (TIF) [file pbio.3002929.s006.tif]
