# Supplementary material for: Axons compensate for biophysical constraints of variable size to uniformize their action potentials
Source: PLoS Biol. 2024 Dec 2;22(12):e3002929. doi: 10.1371/journal.pbio.3002929 (PMC11637306; doi:10.1371/journal.pbio.3002929)
Supplement: S1 Table — Mean and median data with SEM and Q1–Q3 ranges are shown in figures together with individual data points. (PDF) [file pbio.3002929.s007.pdf]

				Student's t test			Norm.			Mann-Whittney U test		
		**mean**	sem	t	DF	p	test	**median**	Q_3-Q_1	U	Z	p
Axon diameter Fig. 1C	sMF	0.785	0.035	20.235	132	$2.8e^{-42}$	$6.8e^{-11}$	0.72	0.41	4345	9.824	0
	LMFB	3.879	0.177					3.58	1.58			
M capacitance Fig. 1D	sMF	0.187	0.019	10.4	109	$5.9e^{-18}$	$9.1e^{-8}$	0.149	0.119	2918	8.436	0
	LMFB	0.893	0.056					0.795	0.605			
input resistance Fig. 1D	sMF	3.76	0.223	-2.51	110	0.013	$7.9e^{-7}$	3.474	1.726	1073	-2.79	0.0053
	LMFB	3.054	0.178					2.684	1.534			
M time constant Fig. 1D	sMF	38.78	1.97	-2.3	108	0.023	$2e^{-4}$	37.00	19.85	1170	-1.92	0.055
	LMFB	33.38	1.39					31.16	14.51			
AP HW raw Fig. 1F	sMF	0.376	0.028	0.853	27	0.401	0.013	0.365	0.165	111	0.517	0.605
	LMFB	0.344	0.016					0.356	0.073			
HW corrected Fig. 1F	sMF	0.215	0.012	-0.236	27	0.815	0.08	0.202	0.071	79.5	-0.855	0.392
	LMFB	0.219	0.008					0.216	0.040			
AP amplitude Fig. 1F	sMF	58.54	3.8	-0.422	27	0.676	0.877	58.54	22.11	89	-0.427	0.669
	LMFB	60.91	3.54					61.98	22.37			
AP ampl corr Fig. 1F	sMF	79.25	3.49	-1.083	27	0.289	0.55	76.54	24.85	71	-1.236	0.216
	LMFB	85.13	3.94					83.73	17.84			
AP area Fig. 1G	sMF	14.77	0.54	1.214	112	0.227	0.883	14.64	6.42	1838	1.356	0.175
	LMFB	15.52	0.35					15.56	4.02			
VSD1 AP HW Fig. 2C	sMF	0.348	0.009	1.467	20	0.158	0.442	0.345	0.020	78	1.116	0.264
	LMFB	0.364	0.007					0.361	0.048			
VSD1 AP area Fig. 2C	sMF	23.48	1.67	0.909	20	0.374	0.222	21.91	7.75	74	0.854	0.393
	LMFB	25.73	1.82					25.97	9.93			
VSD2 AP HW Fig. 2D	sMF	0.313	0.02	1.149	32	0.259	0.134	0.316	0.093	173	0.983	0.325
	LMFB	0.345	0.019					0.373	0.159			
VSD2 AP area Fig. 2D	sMF	20.62	1.83	1.473	33	0.15	0.031	20.67	13.71	194	1.374	0.169
	LMFB	24.34	1.74					25.08	13.92			
VSD AP HW Fig. 2E	filo	0.379	0.025	-1.108	41	0.274	0.846	0.371	0.123	178	-1.276	0.202
	main	0.418	0.026					0.423	0.164			
VSD AP area Fig. 2E	filo	20.61	1.65	-0.314	41	0.755	0.223	22.10	12.17	219	-0.279	0.78
	main	21.4	1.92					20.71	15.10			
I_{Na} local Fig. 5A	sMF	-1.506	0.22	-0.689	28	0.496	$2.4e^{-4}$	-1.339	1.115	93	-0.614	0.539
	LMFB	-1.771	0.338					-1.546	0.995			
I_K local Fig. 5A	sMF	0.897	0.105	-2.07	28	0.047	0.011	0.806	0.698	57	-2.138	0.033
	LMFB	0.581	0.099					0.445	0.434			
I_{Na}/I_K ratio Fig. 5A	sMF	1.723	0.151	4.544	28	$9.6e^{-5}$	0.048	1.571	1.015	189	3.408	0.001
	LMFB	3.07	0.285					3.013	1.703			
I_{Na} density Fig. 5C	sMF	-56.32	11.72	-0.83	91	0.408	$8.4e^{-14}$	-17.88	63.31	1061	-0.15	0.88
	LMFB	-44.4	8.37					-26.00	45.43			
I_K density Fig. 5C	sMF	205.9	27.4	2.464	91	0.0156	$3.7e^{-10}$	137.3	200.6	1359	2.132	0.033
	LMFB	127.6	16.5					98.6	120.2			
I_{Na}/I_K ratio Fig. 5C	sMF	0.271	0.031	1.847	91	0.068	$2.8e^{-7}$	0.205	0.314	1360	2.14	0.032
	LMFB	0.358	0.036					0.291	0.251			
I_{Na} density, DTX Fig. S6B	sMF	-19.31	2.77	0.824	20	0.42	$3.8e^{-5}$	-12.11	17.19	59	0	1
	LMFB	-14.07	4.9					-13.26	8.05			
I_K density, DTX Fig. S6B	sMF	66.48	16.44	-0.273	20	0.788	$1.5e^{-4}$	39.5	94.6	58	0	1
	LMFB	59.43	20					43.0	40.7			
I_{Na}/I_K ratio, DTX Fig. S6B	sMF	0.353	0.058	0.533	20	0.6	0.002	0.283	0.221	65	0.401	0.689
	LMFB	0.309	0.053					0.273	0.234			

S1 Table | Statistical results of comparison of passive and AP properties of sMFs and LMFBs. Mean and median data with s.e.m. and Q1-Q3 ranges are shown in figures together with individual data points.
