# Supplementary material for: Axons compensate for biophysical constraints of variable size to uniformize their action potentials
Source: PLoS Biol. 2024 Dec 2;22(12):e3002929. doi: 10.1371/journal.pbio.3002929 (PMC11637306; doi:10.1371/journal.pbio.3002929)
Supplement: S2 Table — Mean data are shown in Fig 3C. (PDF) [file pbio.3002929.s008.pdf]

**Posthoc Bonferroni test on AP area of different cell types**<br>**P values**<br>*One-Way ANOVA, DF:4, mean^2: 446, F = 27.2, p > 9.95*10^{-17}*					
n =	**LMFB**<br>*64*	**sMF**<br>*50*	**SuMa**<br>*16*	**MCa**<br>*7*	**CB1Ra**<br>*8*
**LMFB**		1	$9.5*10^{-15}$	0.0024	0.59
**sMF**			$8.9*10^{-16}$	$5*10^{-4}$	0.2
**SuMa**				0.238	$4.6*10^{-4}$
**MCa**					1
**CB1Ra**					

S2 Table l Statistical results of entire population (ANOVA) and pairwise (Bonferroni) comparison of AP area data of different axon types. Mean data are shown in **Fig 3C**
