# Supplementary material for: Axons compensate for biophysical constraints of variable size to uniformize their action potentials
Source: PLoS Biol. 2024 Dec 2;22(12):e3002929. doi: 10.1371/journal.pbio.3002929 (PMC11637306; doi:10.1371/journal.pbio.3002929)
Supplement: S3 Table — Linear fits are shown in Fig 3D. (PDF) [file pbio.3002929.s009.pdf]

**Linear fit statistics**<br>adjusted R^2 values<br>*ANOVA p > F values*<br>Intercept / **slope**	AP area<br>vs.<br>structure capacitance	AP area<br>vs.<br>membrane time constant
**all MF**	0.017<br>*0.09*<br>14.5 / **1.07**	
**LMFB**	0.02<br>*0.14*<br>14.4 / **1.17**	-0.016<br>*0.84*<br>15.7 / **-0.006**
**sMF**	-0.021<br>*0.854*<br>14.8 / **-0.8**	-0.019<br>*0.77*<br>15.2 / **-0.012**
**SuMa**	-0.076<br>*0.786*<br>25.4 / **1.17**	0.121<br>*0.12*<br>22.1 / **0.138**
**MCa**	-0.248<br>*0.936*<br>18.2 / **1.86**	-0.239<br>*0.859*<br>20.6 / **-0.109**
**CB1Ra**	-0.166<br>*0.967*<br>18.3 / **0.71**	-0.064<br>*0.475*<br>14.9 / **0.164**

S3 Table I Statistical data on the correlation between size and membrane time constant with AP shapes. Linear fits are shown in **Fig 3D**.
