# Supplementary material for: Axons compensate for biophysical constraints of variable size to uniformize their action potentials
Source: PLoS Biol. 2024 Dec 2;22(12):e3002929. doi: 10.1371/journal.pbio.3002929 (PMC11637306; doi:10.1371/journal.pbio.3002929)
Supplement: Attachment — Submitted filename: response to reviewers Brunner sMF.docx [file pbio.3002929.s010.docx]

Reviewer #1:

How do living animals spatially scale properties to maintain a given function across species of different size or, even in the same individuals, in equivalent structures with different size? A straightforward example of this fascinating question is the generation and propagation of electrical signals, in particular the action potential (AP), in neurons. This phenomenon is governed by complex non-linear rules involving activation of ion channels and passive properties that depend on the morphology of the neuronal structures. Given these governing rules, the simple linear scaling of active properties does not compensate for size changes (as the authors confirm in Fig. S1), suggesting either a feedback providing a precise "genetic tuning", or a functional compensation at the level of ion channel scaffolding. However, tackling the problem experimentally is normally far beyond the available possibilities.

The work by Brunner is an original, elegant and ambitious contribution in which this problem was addressed, may be for the first time, in a very rigorous manner. Using the specificity of mossy fiber (MF) axons, including in the same structure small MFs (sMFs) and large MF boutons (LMFBs), the authors unambiguously demonstrate using patch clamp (Fig.1) and voltage imaging (Fig.2) that the AP is preserved in the two sites despite the great difference in morphology, a result that can be only understood in terms of compensation of active conductance. Interestingly, in other axonal structures which are more difficult to examine, no correlation between the AP and the size of the axon was found (Fig.3). The study continues by examining short-term AP plasticity (long-term metaplasticity was not investigated) showing that the change in the AP shape during a train is the same in sMFs and LMFBs (Fig.4).

The second part of the work focusses on the ion channel compensation necessary to produce the phenomenon. In agreement with the theoretical prediction, the authors find a smaller ratio between sodium and potassium currents in sMF outside-out patches with respect to LMBF outside-out patches (Fig.5A-C and Table S1). Thus, the authors found that inhibition of Kv1 channels by dendrotoxin-I changes the AP shape differently in sMFs and LMFBs (Fig.5D), but this differential effect was not observed when they applied TEA. The authors conclude that the compensatory effect responsible for AP uniformity is exclusive of Kv1 potassium channels. In my opinion this conclusion is not justified and I explain why below.

Before going into details, I want to clarify that this manuscript should be published after some major revision, but additional experiments are not requested. Rather, the authors should critically revise the text and pay attention to the complexity of potassium channels, as well as the possibility that also calcium channels may be involved in the compensation.

We thank the Reviewer for their valuable insights and helpful suggestions.

*(Line numbers in our responses refer to the manuscript text file with highlighted changes.)*

MAJOR POINTS

The authors state that MFs express Kv1, Kv3 and BK channels, neglecting Kv7 (Martinello et al. (2015); Neuron 85:346-63 ; Martinello et al. (2019) Commun Biol 2:145). The role of these channels can be directly tested because they can be selectively blocked by XE991 or Linopirdine (Brown and Passmore (2009) Br J Pharmacol 156:1185-95). The authors also claim that Kv1 only are responsible for AP fine tuning whereas BK channels "provide the backbone of repolarization". This statement is not correct. BK channels are co-activated by calcium and depolarization and they principally couple to a calcium source because they have a low affinity for calcium (Berkfeld et al. (2006) Science 314:615-20). In agreement with this, it was demonstrated that BK channels operate close to the peak of APs both in axons (Filipis et al. (2023) J Physiol 601(10):1957-79) and dendrites (Blömer et al. (2024) Front Cell Neurosci 18:1353895). Also, the role of BK channels can be tested using the selective inhibitor Iberiotoxin. Overall, the evidence provided by the author suggests that Kv1 channels contribute to the compensation, but it does not support the claim that this contribution is exclusive.

We thank the reviewer for highlighting the question concerning the diversity of axonal potassium conductances, and our mistake with the description of the rationale of these experiments. We apologize for omitting other potassium channels in the discussion and incomplete description of the available knowledge about BK and Kv3 channels.

In agreement with the rationale outlined by the Reviewer, and consistent with previous results on mossy fibers (Alle 2011), we stated that both Kv3 and BK channels are present in these axons. Because of the larger contribution of Kv3 to single action potentials, we focused on comparing these channels with Kv1 in terms of their size-dependent contribution to single action potentials. We completely agree with the suggestions of the Reviewer and now clarify in the manuscript that Kv1 is probably not the only mechanism that maintains uniform axonal APs, especially during more complex activity patterns. The aim of the comparison of Kv1 and Kv3 contributions was to test (1) whether there are any potassium conductances whose functions correlate with axon size, (2) and whether all single AP-relevant potassium channels follow similar rules regarding the axon size. We also discuss Kv7 channels in this context and in relation to their major contribution to mossy fiber signaling.

We would like to emphasize that our mechanistic analysis focused only on single APs, not trains. This is important, because it is known that the contribution of different potassium channels changes during more complex activity patterns. Specifically, it has been shown before that the repolarization of single APs is dominated by Kv3 in large mossy fibers (Alle, Kubota, Geiger 2011). However, BK channels, which are also present at high density in these axons, increase their contribution in an activity-dependent manner. We explicitly mention this aspect in the text.

With the experimental comparison of Kv1 and Kv3 functions in MFs, we demonstrated that there is at least one voltage-dependent mechanism whose contribution correlates with the axonal size. Whereas the Kv3 channel serves as an example for size-independent contribution. These results align with previous findings that identified Kv3 as one of the main mediators of AP repolarization in mossy fibers (along with BK channels).

We comprehensively modified the text:

- To highlight the potential roles of additional channels that contribute to APs of mossy fibers (see abstract, main text at lines 203-209, 221-224, 278-281) and to discuss differences in the molecular mechanisms of axonal targeting of Kv7, BK, Kv3 and Kv1 channels (see lines 284-286).
- To clarify that we used Kv1 and Kv3-specific interventions to demonstrate the different size-dependent regulation of AP-relevant potassium channels. The contribution of at least one example, Kv1, changes with the size of the axon, whereas others, such as Kv3, have a consistent contribution regardless of the axon size. We avoid suggesting that Kv1 is the exclusive mediator of size-dependent regulation of AP repolarization (lines 221-224, 281-283, 299-303, abstract).
- To explicitly state that our mechanistic analysis focused on single APs, and that the contribution of different potassium channels changes during trains and complex firing patterns (lines 204-205, 209, 223, 278-280)

We greatly appreciate the precise suggestions of the Reviewer, and we believe that the modifications in the text now provide a more accurate interpretation of the differential contribution of various potassium channels.

It is also not convincing that the compensatory mechanisms are only due to potassium channels. If one looks at traces in Fig. 4E, the changes in AP shape include a decrease in the peak and a widening of the duration. The decrease in the peak should be mainly due to a partial lack of recovery from inactivation of sodium channels whereas the widening should be due to a decrease of potassium current during repolarization. In a simple scheme involving only sodium and Kv1 channels during the peak, an equal decrease in sodium current at sMFs and LMFBs without any change in Kv1 would lead to an equal change in the ratio between sodium and potassium currents, resulting in the preservation of the AP shape at the two sites. However, also potassium (and calcium) channels are affected during the train and it seems unlikely that the tuning of Kv1 only at the different sites can maintain the AP shape uniformity. One should also think that axons are subject to long-term plasticity characterized by changes in ion channel densities. In my opinion, it would be more plausible the hypothesis that multiple channels, operating in synergy, compensate for size variability. Multiple channels can assemble in sub-micron structures guided by scaffolding proteins and auxiliary proteins, which would lead to concomitant regulation. The authors should revise the Discussion. The idea that the homeostatic regulation of a single channel would be simpler and more economic for the system might not be true because only one channel would limit the flexibility that is required during the life of the axon. In contrast, the concomitant regulation of multiple channels that are physically and functionally coupled would provide ways to adapt more efficiently to the complexity of physiological signaling.

Thank you for this suggestion. We have now revised the text regarding rise time and the rationale of focusing on the AP decay (lines 73-74, 81-83, 86-89) and discussed the potential contribution of other channels (see our response to the previous question (lines 203-209, 221-224, 278-281). Furthermore, we generally toned down the conclusions (see deleted sections).

Please note that the rise time of the APs in the raw recordings is the most vulnerable for instrumental filtering. Therefore, we generally avoided drawing conclusions from the measurements of AP rise. Due to the necessity of using small pipettes (which were similar in sMF and LMFB recordings), the voltage signals are inevitably filtered affecting the fastest components the most. Please also see our response to Reviewer #3 concerning the integral area measurements of APs. We would like to additionally highlight that Fig 4E shows APs during trains, during which the upstroke is expected to be significantly affected. As explained above, due to the complex contributions of multiple conductances and biophysical changes during repetitive firing, we generally avoided making claims about the mechanisms of APs during trains, and focused only on single APs (lines 204-205, 209, 223, 278-280).

Our simulations, which allowed us to bypass the effects of instrumental filtering, detected similar INa in small and large mossy fiber boutons, and the IK/INa ratio was consistently higher in smaller boutons. Outside-out patch measurement of INa densities is not affected by these instrumental limitations and allow for the direct comparison of distinct compartments. Our comparison of INa density in small and large MF boutons showed no difference suggesting that at the level of boutons INa is similar. However, it should be noted that sodium channels are expected to accumulate at narrowly restricted membrane surfaces just before diameter widening or bifurcation points to compensate for the capacitive load/sink. The faster speed of the rising phase requires spatially restricted boost compared to the slower decay phase. However, outside out patch recordings do not have the necessary spatial resolution to identify these tiny INa hotspots within axonal compartments. Therefore, sodium channel inhomogeneity can also contribute to maintaining uniform AP, while maintaining their primary function: reliable propagation of APs. Please see our new analysis showing the location-dependent correlations of rise and decay at different (revised SFig.S1 and Fig.1C). We also added a sentence to the discussion to provide an outlook for future studies about the precise subcompartmental localization of channels (lines 291-293)

**Reviewer #2:**

This paper shows convincingly that the action potential in the axons of mossy fibers has nearly the same shape in parts of the axons with small boutons as in the large boutons, and that in outside-out patches from the small boutons, there is a larger contribution from Kv1 potassium channels relative to sodium current. The authors argue that this suggests a general principle that ion channels are regulated in parts of axons with different sizes to "uniformize" action potentials.

From a technical standpoint, this is a remarkable paper. The initial papers from the Jonas and Geiger labs of recordings from mossy fiber boutons were amazing feats of electrophysiology, and the recordings here from even smaller structures reach a new level of experimental achievement. These are very difficult experiments, and they are done with exceptional attention to detail - for example making a correction for the capacitance pf the recording pipette - and with exceptional thoroughness. The amount of data and the technical sophistication and completeness of the data reach a level that has rarely been achieved in any electrophysiology paper. The experimental conclusions are very convincing. It is a major contribution to the literature on action potentials.

We thank the reviewer for the supporting words about our work and helpful suggestions.

*(Line numbers in our responses refer to the manuscript text file with highlighted changes.)*

That said, I was not convinced by the conclusion that the authors have discovered a general principle of action potential uniformity. It is certainly interesting that there is a different combination of channel densities in different regions of the axons, but it does not seem convincing to me that the reason for this is that action potential shape needs to be kept exactly uniform. The synapses at the small and large boutons are different in many ways, e.g. in size of pre-synaptic and post-synaptic region, and small differences in the shape of the presynaptic action potential would seem to be a very minor factor in determining the overall pre- to post-synaptic function. I find it hard to believe that if it were possible somehow to adjust the channel densities so that they were uniform along the axon and there were then small differences in action potentials that overall circuit function would be affected very much. For that reason, I was unconvinced by the repeated statement, starting in the title, that the difference in channel densities represents an "active" compensation, as if there must be some sort of feedback mechanism forcing the density of Kv1 channels to be such that action potential width is similar. It seemed to me that this wording ascribes a purposeful agency to axon design that may not be warranted. I would offer that as one reader's reaction and leave it to the authors whether to keep this wording, which to me seemed a bit provocative.

We agree that the contribution of axonal AP shape to release is smaller than that of the robust synaptic mechanisms, however, it does have consistent effects. For example, as shown previously (e.g. Borst 1999, Pattillo 1999, Geiger 2000, Bischofberger 2002, Kawaguchi 2015, Begum 2016, Martinello 2019), activity-dependent broadening of axonal APs changes axonal calcium influx and synaptic release. Furthermore, axonal AP shapes are cell type-specific, and they are different from somatic APs. If AP shapes were allowed to change with bouton size, it would be difficult to manifest the contribution of these activity- and cell-type-dependent contributions, because the size-induced AP shape changes are in the same range as those predicted by previous theoretical works and also confirmed by our simulations with homogeneous conductances. To clearly highlight these effects, we now include a simpler version of the hypothesis-testing initial modelling results in the main figure, and also extended these initial simulations to highlight the contribution of size-dependent AP variabilities and their functional consequences (see new panels in Fig S1). We also clarified the specific aims of this study in the introduction and after the initial simulations (lines 51-54, 81-85).

We thank the reviewer for highlighting our mistake that we used “active” in this context, which could be interpreted as an “intention” of axons to use uniform APs. We revised the text to avoid this issue and to better explain the relevance of size-dependent AP shape changes (see Title, Abstract, title of paragraph at line 187, title of Fig 5, and lines 55, 57, 219-224, 231-236 in the main text). In fact, our original motivation for this project was to explore how much contribution of size-dependent AP variability has on axonal output functions. The simulations indicated a significant effect, even though their range is smaller than some of the conventional synaptic plasticity mechanisms. Therefore, it was surprising in our initial recordings that even the ten-fold size difference of mossy fiber diameter is not reflected by the AP shape. We then verified this observation with other approaches and explored the underlying mechanisms. We did not want to be provocative with the discussion of these results, rather, we wanted to find a potential explanation for why axons invest energy into mechanisms that maintain uniform APs.

We agree with the reviewer that the original version of the manuscript was not clear about these motivations, and we extensively revised the text to determine clearer aims and to clarify the conclusions (*see* abstract, and lines 51-54, 86-88, 227-234, deleted sentences: lines 184-185, 219-221, 234-236). We also revised the theoretical explorations of the original version of the discussion.

We added a sentence to the Results to highlight that during Kv1 inhibition, the APs exhibited a size dependence similar to that expected from the simple biophysical model (lines 213-215), as this is an important additional argument for the contribution of active conductances for size-independent uniform APs. Rearrangements of panels in the main and supplementary figures also facilitates the direct comparison of the phenomenon.

For example, an alternative interpretation of the results could be simply that the density of Kv1 channels is higher in the non-bouton parts of the axon than in boutons because of factors in bouton formation having nothing particularly to do with fine-tuning AP width. If so, there is unlikely to be an all-or-none transition between "axon" properties and "bouton" properties on the scale of a micron (or even less, since some axons are as small as 0.1 micron), it may just be that most of the membrane in small boutons is less different from normal "axon" properties than that in larger boutons. In fact, the recordings from "SMF"s included recordings from the main axon as well as small boutons. So an alternative interpretation could be that bouton membrane has a lower density of Kv1 channels than axon proper -there are obviously many differences between bouton membrane and axon membrane, including pre-synaptic release machinery, probably uptake transporters, calcium channel dendity, etc - having nothing to do with a requirement to force the AP width to be uniform between boutons and axons.

Thank you for this suggestion. We have revised the discussion to include alternative explanations (lines 301-303).

We also agree that electrophysiological recordings cannot distinguish synaptic and non-synaptic sites. It was not possible to identify synapses in these experiments using posthoc immunolabelling because of the pipette. This motivated us to analyze VSD imaging experiments anatomically (SFig.2), which not only allowed us to distinguish synaptic and non-synaptic sites, but also provided a comparison of APs at high spatial resolution. Again, our motivation for these experiments was the same as that of the Reviewer; it was difficult to interpret initially that the APs are the same in every part of these axons regardless of their distinct functions. This further motivated us to understand the underlying mechanisms in the later part of the work. We have revised the text to better highlight these experiments (lines 145-148).

Although the paper is in general very well-written, there was one notable omission in referring to the literature on variability of action potential shape in cells bodies, axons, and presynaptic terminals -the Hoppa et al. paper (Neuron. 84:778-89, 2014) showing that the action potential size is much smaller in small presynaptic boutons than in cell bodies, as a consequence of increased density of Kv1 and Kv3 channels, and suggesting this is important to allow increased sensitivity of transmission to changes in AP shape.

Thank you for the suggestion. We now added this paper to the manuscript with additional discussion about the relevance of their findings.

The idea that the AP shape is **actively** constrained at presynaptic terminals seems at odds with the idea that there must be uniformity of action potential shape. Of course, in both that paper and this manuscript, the essential information is the experimental observations of AP size and shape in different cell regions, and the speculation of particular reasons this may be important remains hard to prove.

We agree that using the word “active” could be misinterpreted in the original version of the manuscript. We intended to highlight that active conductances contribute to the compensation of the passive, biophysical consequences of different axon size. We have revised the manuscript at several points to be clear about the “active” compensation. For example, we changed the title by removing this potentially ambiguous expression, revised the text to provide more precise conclusions (Title, Abstract, titles of Fig 5 and relevant paragraph, and lines 55, 57, 219, 235).

By using “active” we did not mean a dynamic “intent” of axons, but we wanted to refer to voltage-dependent non-linear mechanisms as opposed to linear, passive mechanisms. Thank you for highlighting this mistake.

Altogether, the authors are to be congratulated on a technically remarkable tour-de-force description of action potentials in axons and boutons. I was not convinced that the data justifies the conclusion that there is an "active" feedback process that is designed to ensure uniformity of action potentials. If it were my paper, I would soften this conclusion. However, I believe that should be the authors' choice.

We hope that the revisions clarify the conclusions, and the Reviewer maintain their positive opinion about our work.

**Reviewer #3:**

In the paper by Brunner and colleagues, the researchers present findings from both electrical and optical measurements made from hippocampal axons. They explore the variability of action potentials (APs) within axons, particularly in relation to the diverse geometrical properties of subcellular domains, and how these APs differ from one axon to another. The authors' successful identification of various axon types is noteworthy, and they have amassed a unique and invaluable dataset. The electrophysiological experiments are well-conducted, and the computational model is meticulously developed, providing ample and multiple lines of evidence to convincingly argue that APs travel uniformly within a single type of axon. However, my primary reservation lies in the ambiguity surrounding the so-called 'theoretical rule'. Both the theoretical foundation and the main conclusion could benefit from a more thorough biophysical explanation, and there is a pressing need to further our understanding of how Kv1 interacts with passive membrane properties.

We thank the reviewer for the supporting opinion about our work and helpful suggestions.

*(Line numbers in our responses refer to the manuscript text file with highlighted changes.)*

1. Supplemental figure S1 is in my opinion a key figure to understand the scientific rational and deserves to be a main figure. The authors show that with variable axon diameters but uniform voltage-gated conductances the cable properties underlie local AP variability. The description this result section is muddled (lines 62-74) and the authors only mention there is "variability" but without offering testable predictions or a detailed explanation for the biophysical properties contributing to the AP. It seems to me that the LMFB exhibit both a fast rise and fast decay rate of the membrane potential, illustrated by the linear relationship between rise- and decay rates. In the face of uniform voltage-gated sodium and potassium conductances, how can a small capacitance domain, both slowing the AP up- and downstroke rate? If a 2.5 kV/s upstroke is associated with a -0.5 kV/s decay rate there may be a passive contribution for normalizing the AP area; an increased depolarization rate simply activates more efficiently potassium conductances, speeding repolarization. How is Kv1 slowing the upstroke of the AP? More detailed insights into the role of the electrotonic properties to the AP in a uniform model should be explored and properly explained. For example, the authors could plot conduction velocity or the dV/dt decay rate as a function of somatic distance to better show the rule how bouton size shapes the local AP. The parameter AP area is ambiguous and not helpful to explain the biophysical basis.

Thank you for these helpful comments, we have made several changes to the figures and text to clarify these issues.

We revised the figures and now included a short version of the model prediction in the main Figure1.

We also provide a new analysis comparing the upstroke and decay of APs along the realistically simulated mossy fiber axon (SFig.1C). Specifically, to clarify the correlation between the rise and decay phase of the APs, as suggested by the Reviewer, we analyzed parameters of local APs from several perspectives. We explored the correlation between the local AP rise/decay and the axon size. We also show that the correlation between the rise and decay is generally linear, as implied by the reviewer. However, there is a significant variability in the rise/decay data of individual APs, as shown in the data now in SFig.1C. The variability can be caused by multiple factors. First, the rise - being faster - is affected by a smaller membrane area than the slower decay phase. More importantly, the propagating APs are affected by the capacitance mismatch caused diameter expansion and tapering at boutons. Thus, the correlation between the rate of voltage changes and size is not linear or monotonous

We clarified the results about the prediction and hypothesis forming models. Specifically, we improved the description of the biophysical contribution of axon size variability and consequential capacitance mismatch (lines 69-74,81-88) in addition to the relatively simple and straightforward effects of the local axon size.

In our models, we did not simulate Kv1 conductance specifically, only a homogeneously distributed delayed rectifier, which - as the reviewer pointed out - did not differentially affect the rise of the AP. Instead, the upstroke was affected by the differences in passive membrane properties due to different axon size and the size/capacitance mismatch. The aim of the simple simulations was to explore the extent of these biophysical effects on AP shape and output.

Furthermore, we would like to highlight that when Kv1 channels were inhibited the axon size and AP shape showed a similar correlation as predicted by simulations using homogeneous conductances. This observation argues for an active role of Kv1 channels in the maintenance of uniform axonal APs, and against the passive contribution of changes in depolarization rate and passive normalization of AP area. Upstroke was not affected by Kv1 function. We added additional text to clarify this observation (see lines 209-213).

We thank the reviewer for raising the point about passive normalization. Indeed, a faster AP rise with a larger AP peak can normalize AP area by more effective activation of Kv channel. However, our pharmacological results are contrary to this alternative hypothesis since the AP area would be uniform even in the presence of dendrotoxin in this case. We highlight this argument at lines 213-215.

Lastly, we clarified the advantage and necessity of using AP area measurements in small pipette recordings. We used AP area measurements because they are barely affected by instrumental filtering caused by the small pipettes needed for these recordings. We employed the same parameter (together with others as suggested) to obtain comparable data. Furthermore, the potential limitations of AP area measurements and pipette filtering effects motivated us to design the complex instrument+biology simulations that allowed us to isolate instrumental effects from biological phenomena (see below our response and changes regarding the technical aspects of the AP area measurements).

2. The tentative conclusion-like arguments (last sentence p.3) on anchoring of Kv1 channels is wildly speculative. Furthermore, about 50% of the discussion is about anchoring of Kv1 channels (PSD 93, ADAM and Caspr2, p. 8-10) and the authors even adventure into the literature on RNA editing of Kv1. However, data to substantiate the differential distribution of anchoring is completely absent and no experimental examination has been conducted to uncover an axon size-dependent clustering and its impact on action potentials. Rather than conjecture, the authors should provide a robust biophysical explanation of the results and cautiously elucidate how a domain with small capacitance leverages Kv1 to affect the properties.

We added new text to provide a more detailed explanation of the biophysical circumstances of axonal AP signaling and to give accurate context to our findings (abstract, lines 69-74, 81-88, 228-231, deleted: 184-185, 219-221, 294-298). We also shortened the implications of the putative molecular mechanisms that could mediate clustering of potassium channels based on the size of the axonal structures. Nevertheless, we believe that it is an interesting correlation that Kv1 channels that have size-dependent contribution to the repolarization of MF APs are associated with different anchoring pathways than Kv3 channels, which have similar contributions regardless of axon size. Therefore, we believe that our findings can motivate new molecular biology studies to clarify the components that potentially link axon size to channel densities (lines 244-255, 284-293). We also revised the discussion regarding the contribution of Kv1 to APs (lines 221-224, 231-234, 281-283)

Minor comments

The accuracy of citing the literature needs to be improved. Just one example, refs. #46 and #47 are not directly comparing large versus small boutons as claimed (lines 185, 249). The publications use EM and qualitatively reveal specific ion channel isoform distribution across compartments.

We revised the text to be more specific about previous anatomical findings (see lines 206-208, 304-309).

"Speed of passive voltage changes was also slower in sMFs" (p. 4). The membrane properties were determined in normal extracellular recording solution and thus not passive. "Resting membrane properties" is the correct terminology.

Corrected (lines 97-98).

Please explain why the integral of the AP is a better parameter. For Kv-related AP properties the authors need to convert the voltage-time data into the first temporal derivative.

We show now the dV/dt data in SFigure 1C.

We have added a clearer description about the integral area measurements (see lines 102-106, 118-119). We also added new data about the reliability of AP area measurements (see **S1F Fig**). Specifically, we used the corrected AP data to show that despite the significant filtering effect the AP area measurements of the raw signal reliably predicted AP kinetics, whereas the more classical half-width measurements were heavily affected.

We also revised the relevant parts of the Methods (lines 402-405)

"This suggests that AP uniformity is a fundamental feature of axonal APs, beyond they plasticity and passive biophysics" (p. 9). Hard to understand and an awkward sentence. Please rewrite.

We removed this sentence.

"the amount of Kv1 channels correlate with bouton size" (p. 9). Correlation implies continuous variables are plotted against each other. Where are these data?

Corrected (lines 282-283).
