# Supplementary material for: Axons compensate for biophysical constraints of variable size to uniformize their action potentials
Source: PLoS Biol. 2024 Dec 2;22(12):e3002929. doi: 10.1371/journal.pbio.3002929 (PMC11637306; doi:10.1371/journal.pbio.3002929)
Supplement: Attachment — Submitted filename: R2 Response to reviewers.docx [file pbio.3002929.s011.docx]

***Reviewer #1****: The authors addressed my previous points satisfactorily, in particular by toning down some conclusions that are not supported by the current results. In my view the manuscript can be published in the present form.*

We thank the Reviewer for their supportive opinions on the revised version of the manuscript.

_________________

***Reviewer #2****: The revisions have addressed all the major issues raised by the referees. Although the manuscript is generally written clearly, there were a few cases where the phrasing seemed unclear.*

We thank the Reviewer for their supportive opinions on the revised version of the manuscript and for the helpful suggestions, all of which are included in the manuscript now.

*54 " Our results provide strong evidence for the maintenance of size-independent AP uniformity within single axons of various types. Among them, hippocampal mossy fiber boutons use Kv1 channels for this compensation of the consequences of their variable size, consistently with the distinct anchoring mechanisms and unusual distribution of these channels."*

*The second sentence might be phrased slightly more clearly as " In hippocampal mossy fiber axons, Kv1 channel density varies in boutons of different sizes in a manner that compensates for changes in AP shape otherwise expected from capacitative and resistance effects. The varying density of Kv1 channels may be related to distinct anchoring mechanisms and unusual distribution of these channels."*

Done (line 56-60, in the text version with tracked changes)

*193 " Among axonal Kv channels, Kv1s are known to be responsible for fine-tuning APs for specific functions of various axons [4, 6-9, 13, 14, 40, 42], whereas Kv3 and BK channels usually provide the backbone of repolarization in mossy fibers [43] and Kv7 channels set axonal excitability that also influence spiking properties [44]. However, their contribution changes during complex firing patterns"*

*The second sentence could be interpreted as referring to the contribution of Kv7 channels. I think the meaning might be clearer as "The contribution of different Kv channels changes during complex firing patterns".*

Done (line 199-200)

_________________

***Reviewer #3****: The manuscript has been greatly improved and offers more insight. While the biophysical basis has been clarified better I have still a few minor concerns.*

We thank the Reviewer for their supportive opinions on the revised version of the manuscript and for the helpful suggestions, which we address below.

*About the passive normalization the authors state their "pharmacological results are contrary to this alternative hypothesis since the AP area would be uniform even in the presence of dendrotoxin in this case. We highlight this argument at lines 213-215" I can't find such explanation in the text. The authors should offer academic explanations how to explain the contradiction between theory and experiment.*

Thank you for this suggestion. We revised the description and interpretation of these results about the size-dependent APs measured in DTX conditions (see lines 210-214, in the text version with tracked changes).

*It would help showing directly whether the smaller boutons have locally larger membrane time constants in the face of constant channel densities. FigS1A lists claims which are not shown but assumed.*

Data showing slower membrane time constants measured at smaller and larger boutons were already included in the main text (lines 95-96), Figure 1D and also in Table S1.

However, as the Reviewer correctly suggested, the locally measured membrane time constant does not reflect only the local membrane by definition. Thus, locally measured membrane time constant reflects neighboring axon segments depending on the duration of the applied current pulses. As such, in small structures, like axons, it is not possible to measure or simulate the membrane time constant with conventional current pulses. Therefore, our starting point was the simulated expected effects of axon size on AP kinetics (SFig.1 B,C,F), which is both theoretically more reproducible and biologically relevant prediction.

To avoid any potential confusion caused by the features of local membrane time constant, we revised the SFig.1A by removing reference to local membrane time constant. We revised the text (Line 95: we added “measured membrane time constant”, instead of “membrane time constant”). And clarified the legend for Figure 1D.

*Note, some Kv1.x channels have also an impact on the resting membrane properties (e.g. Vm) and so it would be good to report such potentially important roles of Kv1.x channels for the MF axon.*

Thank you for this suggestion. We added the resting membrane potential data in control versus DTX in small and large boutons (see lines 216-220).

*Line 204 "biophysically limited structure". Unclear what the authors mean with this.*

Done (line 208-209)

*Line 284. 'Direct patch clamp recordings are not possible from the myelinated parts of SuM, MC, CB1R axons'. What is the evidence those axons are myelinated? The images in Figures S3-S5 show a high densities of release sites and, as the authors rightly stated above in the manuscript, myelin would limit presynaptic bouton formation.*

We extended and clarified the discussion about the myelinated axonal segments (see lines 296-298).
